# A Systematic Review of Interventions for Demoralization in Patients with Chronic Diseases

**DOI:** 10.1007/s12529-024-10262-w

**Published:** 2024-02-05

**Authors:** Li Dong, Li Li, Yunlian Wu, Xiaoling Zhao, Hui Zhong, Xi Cheng, Lixia Liu, Changxia Cheng, Mingqiu Ouyang, Liande Tao

**Affiliations:** Department of Nursing, The Second People’s Hospital of Yibin, Yibin, 644000 China

**Keywords:** Demoralization, Psychological intervention, Chronic disease, Patients, Systematic review

## Abstract

**Background:**

Demoralization, a significant mental health concern in patients with chronic diseases, can have a large impact on physical symptom burden and quality of life. The present review aimed to evaluate the effectiveness of interventions for demoralization among patients with chronic diseases.

**Method:**

PubMed, Scopus, Embase, and Web of Science were systematically searched. Research on providing interventions to patients with chronic diseases that included quantitative data on demoralization was then systematically reviewed.

**Results:**

Fourteen studies were included, most of which considered demoralization as a secondary outcome. Interventions included evidence-based meaning-centered psychotherapy, dignity therapy, psilocybin-assisted psychotherapy, and others. Ten studies used randomized controlled designs. Six of these investigated evidence-based meaning-centered therapy, and four investigated dignity therapy, showing the best empirical support for these intervention types. Most studies showed significant impacts on demoralization in patients with chronic diseases.

**Conclusion:**

This systematic review provides insights into potential psychological interventions for reducing demoralization in patients with chronic diseases. Randomized controlled designs and adequately powered samples, with demoralization as the primary outcome, are needed to more clearly evaluate its effectiveness.

**Supplementary Information:**

The online version contains supplementary material available at 10.1007/s12529-024-10262-w.

## Introduction

Demoralization is an existential distress syndrome that may occur when one is in an unsettling situation or environment, including chronic diseases (e.g., cancer, acquired immunodeficiency syndrome [AIDS]) that threaten the patient’s integrity of being or health [1, 2]. Frank [3] first used the term ‘demoralization’ in the psychiatric literature, to describe an abnormal mental state resulting from a persistent inability to cope with internal or external stressors with which the individual—and those close to them—expect them to deal, and is mainly manifested as feelings of incompetence, loneliness, and despair. de Figuereido and Frank [4] claimed that demoralization includes distress and subjective incompetence, which coexist when assumptions related to self-esteem do not hold.

In addition to a feeling of subjective incompetence, a demoralized person has lost their ability to look forward with pleasant anticipation. According to Clarke and Kissane’s model, demoralization includes five sub-dimensions: hopelessness or disheartenment, loss of meaning in life, helplessness, sense of failure, and dysphoria [5, 6]. Healthcare providers often ignore or fail to recognized demoralization because it is considered a normal reaction of an individual to their medical condition [6]. Demoralization can reduce an individual’s ability to cope with stressful events, which may lead to a series of adverse health outcomes, such as impaired physical symptoms, restricted social functioning, and damaged quality of life [7, 8]. Therefore, timely recognition of and addressing demoralization is crucial to patient health and quality of life.

Demoralized patients often experience depression, and there is comorbidity between them [9]. Thus, it is important to correctly assess demoralization in patients with chronic diseases. The difference between demoralization and depression is that the core symptoms of the former include loss of hope and meaning for the future, while those of the latter are loss of joy and interest in the present [5, 6]. Further, the major concern in demoralization is subjective incompetence and uncertainty about the applicable direction of action, while that in depression is a decline in motivation, even if the applicable direction of action is known [10].

Scales to accurately measure demoralization have been designed. The 24-item Demoralization Scale (DS) was developed by Kissane et al. [11] and Robinson et al. [12] developed the simplified 16-item DS-II. Both scales are in use in multiple countries as effective psychological measurement tools.

Chronic diseases are characterized by an illness duration of ≥ 3 months and a slow disease progression [13]. Although most patients with chronic diseases never experienced demoralization [14], certain aspects of chronic diseases may trigger negative expectations for the future, making demoralization more likely. A gradual decline in function [15], financial burden [16], and loneliness [17] are common among patients with chronic diseases, contributing to loss of hope and meaning in the future. Demoralization has become an important concept in medical psychiatry over the past two decades [5, 6]. Because demoralization is treatable, diagnosing it as a clinical syndrome refines focus on potential treatment interventions [6]. However, published demoralization intervention studies are limited and most focus on related constructs like distress and depression, including demoralization as a secondary outcome.

Frank [3] argued that demoralized individuals are willing to seek psychotherapy, as they are in a highly suggestible state that interacts with expectations for improvement in this therapy environment. Demoralization thus frequently prompts patients to seek psychotherapy, the effectiveness of which lies in the ability to restore the individual’s morale [18]. Despite several studies concluding that psychological interventions reduce demoralization in patients with chronic diseases, their effect sizes have varied. Some interventions have reported a positive effect on demoralization [19, 20], while others have not [21, 22].

Reducing demoralization in patients with chronic diseases can promote their perception of life’s meaning, value, and purpose, and improve their resilience and ability to cope with negative life events. However, there are limitations to using these findings as an evidence-base for treatment. Because, to our knowledge, there has been no comprehensive review of demoralization interventions in patients with chronic diseases, this was the aim of this systematic review, toward identifying effective strategies for this patient population.

## Methods

PRISMA guidelines were followed throughout, and the population, intervention, comparison, outcome, study design (i.e., PICOS) search method was used. Only quantitative studies were included, to allow their statistical comparisons.

### Eligibility Criteria

#### Participants and Interventions

Studies of patients aged ≥ 18 years with chronic diseases were included. Chronic diseases were characterized by an illness duration of ≥ 3 months and a slow disease progression (e.g., cancer, AIDS) [13]. A study was considered for review if it involved patients with chronic diseases, even if it used alternative terms like terminally ill patients. Various intervention (e.g., pharmacological, psychological, spiritual) studies targeting demoralization were included.

#### Included Study Designs and Outcomes

Due to the limited use of randomized controlled trials (RCTs) targeting demoralization among patients with chronic diseases, pre-post methods with quantitative reporting were also included. Demoralization was defined as a persistent mental state that arised from an individual’s inability to effectively cope with a stressful event. It was characterized by five domains: hopelessness or disheartenment, loss of meaning in life, helplessness, sense of failure, and dysphoria. Studies examining demoralization were included if they used some quantitative measure of demoralization, such as the Demoralization Scale [11] or Demoralization Scale-II [12].

#### Exclusion Criteria

Case reports, opinions, and reviews were excluded. The primary review outcome was demoralization. Studies not published in English were excluded.

### Search Strategy

PubMed, Scopus, Embase, and Web of Science were searched from inception to February 2023. The search strategy (see Supplementary Table 1) was developed for PubMed in consultation with an information scientist, then translated to other databases. OR was used in search strategies to combine keyword synonyms and spellings under the main concepts; AND was used to combine concepts.

### Data Extraction

Two researchers independently screened each article for the inclusion and exclusion criteria. Disagreements were resolved through consensus or involvement of a third researcher. Extracted data included participant characteristics, interventions, outcome measures, sample sizes, and main findings.

### Quality Assessment and Risk of Bias

The Cochrane Risk of Bias Tool was used to assess RCT quality as low, high, or unclear on each of five domains: random sequence generation, deviation from intended intervention, incomplete outcome data, blinding of outcome assessment, and selective reporting. The Cochrane Risk of Bias Assessment Tool for Non-randomized Studies (RoBANS) was used to assess non-RCT quality as low, high, or unclear on each of seven domains: confounding bias, participants selection bias, interventions classification bias, deviation from intended intervention, incomplete outcome data, blinding of outcome assessment, and selective reporting. Two authors independently assessed the risk of bias and resolved disagreements by discussion until consensus was reached for all included articles. Following completion of quality assessment, an overall risk of bias for each study was summarized in the evidence table.

### Analysis

Due to heterogeneity in participants, interventions, and outcomes, overall effect sizes could not be calculated.

## Results

### Summary of Studies

**Fig. 1:**
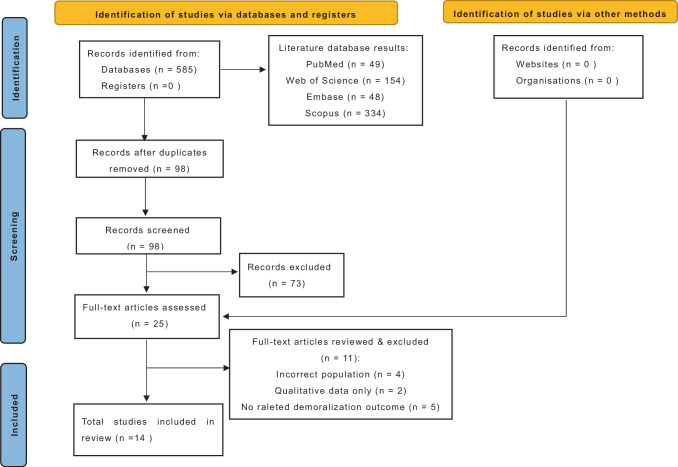
Study flowchart

Fourteen studies met all inclusion criteria (Fig. 1) [19–32]. Study designs, interventions, participants, assessments, main findings, attrition rates, and risks of bias are summarized in Table 1. Table 2 provides intervention duration and interventionist details, and brief intervention descriptions. Most of the included studies were published within 5 years of the search end date.

**Table 1:** Summary of demoralization intervention studies

Author/s	Country	Design	Intervention	Participants	Outcome tools	Findings	Attrition rate (%)	Risk of bias
Sun et al. [19]	Taiwan	Pretest-posttest	Logotherapy	34 patients with breast and gynecological cancer	DS	Reduced demoralization (*p* = 0.000)	9	High
Quilez-Bielsa et al. [20]	Spain	Pretest-posttest	MCP-EC	30 patients with advanced cancer	DS	Reduced demoralization (*p* = 0.005)	7	High
Fraguell-Hernando et al. [23]	Spain	RCT	IMCP-PC	51 patients with advanced cancer	DS-II	Reduced demoralization (despair, *p* = 0.001)	37	High
Kissane et al. [21]	Australia	Pilot RCT	MaP	57 patients with advanced cancer	DS-II	No significant between-groups demoralization differences	11	Low
Rodin et al. [24]	Canada	RCT	CALM	305 patients with advanced cancer	DS	After six intervention months, demoralization reduced among participants with moderate death anxiety (*p* = 0.01)	26	Low
Mehnert et al. [25]	Germany	RCT	CALM	216 patients with advanced cancer	DS	Demoralization not reduced in the intervention group compared with the control group	38	High
Julião et al. [28]	Portugal	RCT	Dignity therapy	80 patients with terminal illness	5 criteria for demoralization	Decreased demoralization incidence (*p* < 0.001)	13	High
Li et al. [27]	China	Pretest-posttest	Dignity therapy	30 patients with terminal cancer	DS	Reduced demoralization (*p* = 0.037)	17	High
De Vincenzo et al. [22]	Italy	RCT	Dignity therapy	111 patients with terminal illness	DS-II	No significant demoralization changes	30	Low
Iani et al. [26]	Italy	RCT	Dignity therapy	35 patients with terminal illness	DS-II	No significant between-groups demoralization differences	45	High
Agin-Liebes et al. [29]	USA	Secondary analysis using pre-post design for RCT	Psilocybin-assisted psychotherapy	15 patients with cancer	DS	Reduced demoralization (*p* < 0.001)	13	Low
Anderson et al. [30]	USA	Pretest-posttest	Psilocybin-assisted group therapy	18 older men with AIDS	DS-II	Reduced demoralization (*p* < 0.05)	5	High
Soto-Rubio et al. [31]	Spain	RCT	Kibo therapeutic interview	64 patients with advanced terminal diseases	SDS	Reduced demoralization (*p* < 0.05)	6	High
Nelson et al. [32]	USA	Pilot RCT	CARE	51 patients with cancer	DS	No significant between-groups demoralization differences	19	Low

*DS* Demoralization Scale, *RCT* Randomized controlled trial, *MCP-EC* Meaning-centered psychotherapy and essential care, *IMCP-PC* Individual meaning-centered psychotherapy-palliative care, *DS-II* Demoralization Scale-II, *MaP* Meaning and purpose therapy, *CALM* Managing Cancer and Living Meaningfully, *SDS* Short Demoralization Scale, *CARE* Cancer and Aging: Reflections for Elders

**Table 2:** Summary of demoralization interventional therapies

Author/s	Unit of intervention	Session numbers and durations	Interventionist/s	Intervention summary
Sun et al. [19]	Individual	4–6 60-min sessions over 12 weeks	Psychologist	Logotherapy to help participants discover life meaning and feel value of self-existence through work, love, and suffering.
Quílez-Bielsa et al. [20]	Individual and group	Phase I: 4 consecutive weekly individual sessions of 45-min each Phase II: 4 consecutive weekly group sessions of 60-min each	Psychiatrist	MCP-EC to promote care and self-care and establish a care plan among patient, family, and healthcare team. Sessions explored care, self-care, responsibility, self-compassion, kindness, and attitude to face advanced cancer.
Fraguell-Hernando et al. [23]	Individual	3 sessions within 4 weeks, each 45–60-min	Psychologist	Psychotherapy to reinforce sense of meaning and purpose in life.
Kissane et al. [21]	Individual	6 sessions, each 60-min	Psychologist	Psychotherapy to develop personalized goals and build coherent patient story about strengths, achievements, goals, and life significance.
Rodin et al. [24]	Individual	3–6 sessions over 3–6 months, each 45–60-min	Psychiatrist	Semi-structured interview exploring domains of symptom management and communication with medical workers, changes in self and other relationships, sense of meaning and purpose, death, and concern for the future.
Mehnert et al. [25]	Individual	6 sessions within 6 months, each 50-min	Psychotherapist	Same as above.
Julião et al. [28]	Individual	4–5 sessions over 1 month, each 30–60-min	Psychologist	Semi-structured interview to improve sense of meaning, purpose, and worth. Sessions transcribed and edited to produce generativity document to bequeath family/friends.
Li et al. [27]	Individual	3 sessions within 1 week, each 30–60-min	Dignity therapist	Same as above.
De Vincenzo et al. [22]	Individual	2 sessions over 2–3 days, each 20–60-min	Psycho-oncologist	Same as above.
Iani et al. [26]	Individual	2 sessions, each 20–60-min	Psycho-oncologist	Semi-structured interview to improve sense of meaning, purpose, and worth. Sessions transcribed and edited to produce generativity document to bequeath family/friends.
Agin-Liebes et al. [29]	Individual	Average 3.2-year and 4.5-year post-intervention follow-ups	Psychiatrist	Psilocybin plus psychotherapy to reduce anxiety, depression, hopelessness, demoralization, and death anxiety.
Anderson et al. [30]	Groups	8–10 sessions, each 90-min	Therapist	Psilocybin-assisted group therapy to enhance personal meaning. Sessions explore meaning, self-compassion, and mindfulness.
Soto-Rubio et al. [31]	Individual	2–3 session, each 1–3-h	Psychologist	Kibo interview to improve mental state, demoralization, and resilience. Sessions explore spiritual needs of patients receiving palliative care.
Nelson et al. [32]	Groups	5 phone sessions over 7 weeks, each 45-min	Psychologist	Group-based therapy to help patients reappraise situation in the context of achieving ego integrity. Therapeutic elements include information-giving and information-receiving, concerns, problem-solving, coping skills training, expression of emotion, and social support.

Fourteen studies were conducted in Spain, Portugal, Italy, USA, Australia, Canada, Germany, and China. The interventions included the evidence-based meaning-centered psychotherapy (*n* = 6), dignity therapy (*n* = 4), psilocybin-assisted psychotherapy (*n* = 2), and other interventions (*n* = 2).

Nine studies restricted inclusion criteria to patients with cancer. The inclusion criteria for the other five were patients with a terminal illness or AIDS. The most commonly used measurements for evaluating demoralization in these studies were the Demoralization Scale (*n* = 7) and Demoralization Scale-II (*n* = 5). One study utilized the Short Demoralization Scale [31], while the remaining study employed five criteria for diagnosing demoralization [28]. Interventions varied from 2–3 days to 6 months. Most included studies were psychological interventions; only two were combined pharmaceutical-psychological interventions. All interventions were psychologist-, psychiatrist-, or psycho-oncologist-administered. Nine studies showed improvements in demoralization outcomes.

### Study Quality

The bias risk assessment results are shown in Table 1. Ten studies were RCTs, and four used pre-posttest designs. Due to the potential pre-existing differences between groups at the beginning of the study, there was a significant risk of bias in studies employing these designs. Additionally, other factors that contributed to a higher risk of bias included challenges faced by participants in adhering to the intervention and remaining in the study over time, small sample sizes, and inadequate methods of allocation concealment.

### Interventions and Study Characteristics

The psychological interventions investigated to date for patients with chronic diseases include evidence-based meaning-centered psychotherapy, dignity therapy, psilocybin-assisted psychotherapy, and others.

#### Evidence-Based Meaning-Centered Psychotherapy

Six studies described evidence-based meaning-centered psychotherapy, designed to enhance a sense of meaning and purpose in life among patients with cancer. Among these, two RCTs showed improvements in demoralization in patients with chronic diseases [23, 24]. Fraguell-Hernando et al. [23] evaluated individual meaning-centered psychotherapy-palliative care (IMCP-PC) effectiveness on emotional well-being and demoralization in patients with advanced cancer. This team used three sessions within 4 weeks. IMCP-PC focused on three domains: (1) self-awareness and meaning; (2) sources of meaning; and (3) finding meaning and a sense of peace. The IMCP-PC group showed improvement in demoralization. Besides, the other RCTs showed that participants assigned to ‘managing cancer and living meaningfully’ (i.e., CALM) had reduced demoralization compared with those receiving usual care, but only at the 6-month follow-up [24].

Two pre-post studies suggest that evidence-based meaning-centered psychotherapy is effective for reducing demoralization [19, 20]. Quílez-Bielsa et al. [20] found that meaning-centered psychotherapy and essential care (MCP-EC) reduced demoralization in their prospective study of 30 patients with advanced cancer. The intervention group received four psychiatrist sessions of either individual or group-based MCP-EC; the control group received usual individual support. In another study, logotherapy was effective for reducing demoralization among patients with breast and gynecological cancer [19].

No significant change in demoralization was observed in either of the other included studies [21, 25]. One study investigated the meaning and purpose (MaP) therapy, which aimed to improve meaning-based coping through life review, with a focus on values, purpose, and determination. However, when compared to usual care, MaP therapy did not show a significant reduction in demoralization among advanced cancer patients [21].

#### Dignity Therapy

Four studies tested dignity therapy in patients with chronic diseases [22, 26–28]; two found benefits for demoralization [27, 28]; and two found none [22, 26]. In Li et al.’s [27] quasi-experimental study, patients assigned to dignity therapy sessions (compared with general visits) showed consistent demoralization improvements through a 1-week follow-up. Julião et al. [28] evaluated the effect of dignity therapy plus standard palliative care in patients with terminal illness, showing a significant decrease in the prevalence of demoralization among those in the intervention group.

Neither of the other RCTs found that dignity therapy plus palliative care significantly improved demoralization compared with palliative care alone [22, 26].

#### Psilocybin-Assisted Psychotherapy

Limited evidence supports the use of psilocybin-assisted psychotherapy for demoralization in patients with chronic diseases [29, 30]. In their analysis of patients with cancer who received this therapy, Agin-Liebes et al. [29] found improvements in self-reported demoralization symptoms at 3.2–4.5 years follow-up. A pre-post psilocybin-assisted group intervention observed significant demoralization differences in older long-term AIDS survivors [30]. Interventions combining psilocybin and psychotherapy thus appear to effectively reduce demoralization in patients with cancer or AIDS.

#### Other Interventions

One study showed that Kibo, a therapeutic interview focused on patients’ spiritual needs, improved demoralization compared with usual care when used with semi-structured interviews of patients with advanced cancer [31].

The novel telephone intervention ‘Cancer and Aging: Reflections for Elders’ (CARE) aims to help older adults better cope with disease and aging needs. One CARE study used a supportive-educative intervention for patients with cancer [32]. Compared with usual care, CARE failed to significantly reduce demoralization in these patients. The impacts of CARE on patients with cancer thus remain uncertain.

## Discussion

This systematic review identified 14 intervention studies that assessed demoralization among patients with chronic diseases. Most research addressed other outcomes (e.g., depression, anxiety) and included demoralization as a secondary outcome. The published interventions included evidence-based meaning-centered psychotherapy, dignity therapy, psilocybin-assisted psychotherapy, and others, with most studies investigating the first two. Most research has tested individual therapy, with more than three sessions.

Heterogeneity in study populations, interventions, and intervention durations preclude clear recommendations based on the effectiveness of demoralization interventions in patients with chronic diseases. Many demoralization-reducing programs have focused on those with advanced cancer. Thus, participant selection bias may be important insofar as those with advanced-stage cancer may experience higher levels of demoralization compared with those with other cancer stages.

Evidence-based meaning-centered interventions were somewhat effective for improving demoralization among patients with cancer, compared with supportive therapy. The purpose of evidence-based meaning-centered psychotherapy is to promote finding meaning and purpose in life [33]. This notion is supported by studies showing that enhancing meaning-making improves multiple outcomes in patients with cancer [34, 35]. Herein, several evidence-based meaning-centered psychotherapeutic techniques were used to reduce demoralization in patients with cancer (i.e., MCP-EC, IMCP-PC, CALM, logotherapy) [19, 20, 23, 24]. Their findings are consistent with the meaning-centered psychotherapy literature, that meaning-centered psychotherapy reduces psychological distress in patients with advanced cancer [36, 37].

One RCT showed that IMCP-PC, an intervention tailored to the needs of patients with cancer in palliative treatment, only improved one of the five demoralization domains: despair [23]. These participants had stage III/IV cancer, and high dropout rates (e.g., from death, health deterioration), which may have biased result accuracy and reliability. That MaP therapy was not effective for patients with advanced cancer may also have been limited by regression to the mean [21]. That CALM therapy was effective for patients with cancer and moderate distress about dying and death only at 6-month follow-up [24] may have reflected a delayed effect. Overall, while these findings support the use of evidence-based meaning-centered psychotherapy for patients with advanced cancer, they provide no consensus on an intervention protocol for this patient population.

Dignity therapy encourages demoralized patients to regain their inner strength to facilitate creation of legacy documents, set future directions, and reduce self-awareness of incompetence [28]. It also prompts patients to express their inner feelings, recall meaningful aspects of life, and impart wisdom to those they love. Inability to express emotions and set goals in the face of illness is a key element of demoralization [22]. Thus, dignity therapy may improve demoralization through patient recall of their most important life events or moments and by establishing a legacy document [28]. Two RCTs with patients with terminal illness suggest that dignity therapy is ineffective for reducing demoralization [22, 26]. It is worth noting that small sample sizes or inadequate blinding may have affected study results. Meta-analyses have indicated that dignity therapy has positive effects on dignity and anxiety among patients with chronic diseases [38, 39]. Nevertheless, its evidence basis in demoralized patients with chronic diseases remains ambiguous [22, 26].

Interventions combining psilocybin and psychotherapy appear to reduce demoralization in patients with chronic diseases [29, 30]. Psilocybin is a psychedelic, historically used therapeutically for neuropsychiatric conditions [40]. It can increase trait openness and cognitive flexibility, and in assisted psychotherapy, promotes rapid, lasting cognitive changes [41, 42]. Despite safety and ethical issues, several studies have shown that psilocybin-assisted psychotherapy can produce lasting mental illness symptom improvement [43–47].

Psilocybin-assisted psychotherapy has also shown potential for reducing demoralization in patients with chronic diseases. Psilocybin-assisted psychotherapy can have long-term benefits for patients with cancer, which are maintained across follow-up up to 4.5 years [29]. The mechanism of psilocybin-assisted psychotherapy may be that it allows participants to establish a new internal framework from which they can flexibly use internal and environmental resources to cope with life stressors. However, a disadvantage of this study’s crossover design is that long-term demoralization improvements cannot be directly attributed to the therapy. Rather, these patients may have experienced naturalistic reduction of demoralization, and resolution of adaptation barriers, during extended survival [29]. Moreover, failure to adequately blind participants to ensure feasibility of group therapy increased study bias [30]. Further, small samples in two studies reduced their statistical power. Larger-sample, adequately blinded trials are needed to confirm the demoralization benefits of psilocybin-assisted psychotherapy in patients with chronic diseases.

The other interventions reviewed herein were Kibo therapeutic interview and CARE [31, 32]. A single study showed impacts with patients with chronic diseases: Soto-Rubio et al.’s study of patients receiving palliative care showed that demoralization improved following Kibo therapeutic interview [31]. This method focuses on the participant’s spiritual needs, based on reflections on their life experiences. Thuné-Boyle et al. [48] suggested that spirituality may help enhance individuals’ self-esteem, sense of meaning, and sense of purpose in life. Meeting the spiritual needs of patients with chronic diseases can improve their quality of life and emotional well-being [49, 50]. Discussing spirituality (including the intrapersonal, interpersonal, and transpersonal dimensions) with participants naturalistically promotes their close attention to spiritual needs and identifies the most important spiritual elements in their lives to alleviate demoralization. Limitations of these studies include the lack of follow-up to show sustainability effects [31] and that participants were primarily white and college-educated, limiting their generalizability [32].

Reducing demoralization can help these patients maintain hope for the future, regain a sense of control over their lives, and respond to stressful events, ultimately improving their quality of life. Demoralization is also negatively associated with suicidal ideation [51]. Thus, timely demoralization interventions may improve overall healthcare and reduce suicide risk in patients with chronic illnesses.

In a systematic review conducted by Wang et al. [52], it was demonstrated that cancer patients who received psilocybin-assisted psychotherapy or psychological interventions experienced a significant decrease in demoralization. While the reviewed study specifically focused on cancer patients, our review expands its scope to include patients with chronic diseases beyond cancer. Consequently, this review offers evidence for intervention approaches targeting demoralization in other chronic/terminal illnesses.

### Limitations

This review was not without limitations. First, most studies included adults with severe illnesses. Assessment of larger, more diverse populations (e.g., adolescent patients, non-severe patients, or caregivers) are needed to test the benefits of these demoralization interventions. Second, most included studies aimed at improving depression, anxiety, and dignity-related distress; only two were designed with demoralization as the primary outcome. Hence, these interventions may not necessarily be effective for demoralization specifically. Thus, caution is needed when interpreting the effects of interventions on demoralization, and their goals and methods should be taken into account. Third, although this review was based on a comprehensive search of the published literature, some studies may have been omitted. Publication bias may also have affected the identified research, impacting the validity and generalizability of our conclusions. Fourth, some included studies’ small sample sizes limited their statistical power. Finally, the interventions were markedly heterogenous (e.g., type, duration). Their limited numbers and lack of available data also meant that a meta-analysis could not be used to quantify their results. We recommend the following implications for future studies in this field: (1) Keep the intervention focus on reducing demoralization rather than other symptoms of psychiatric illness. (2) Increase the sample size to more precisely estimate effects and improve generalizability. (3) Conduct long-term follow-up studies to observe the long-term effects.

## Conclusion

Interest in interventions to reduce demoralization among patients with chronic diseases is increasing. This systematical review of demoralization interventions within this patient population identified methods for developing demoralization-reducing programs. Although evidence-based meaning-centered psychotherapy and dignity therapy were the most common interventions in the review, there were mixed results for its effectiveness on demoralization in patients with chronic diseases, with most findings lacking support. Two rigorous trials have been conducted on Breitbart’s Meaning-Centered Psychotherapy for both groups and individuals with advanced cancer [34, 36]. However, it is important to note that these studies did not include a measure of demoralization. Therefore, further high-quality RCTs are required to specifically investigate interventions aimed at reducing demoralization in patients with chronic diseases.

## Supplementary Information

Below is the link to the electronic supplementary material.

Supplementary file1 (DOCX 19 KB)

## Data Availability

Data are available on reasonable request.
