# Supplementary material for: A Systematic Review of Interventions for Demoralization in Patients with Chronic Diseases
Source: Int J Behav Med. 2024 Feb 5;32(1):1–10. doi: 10.1007/s12529-024-10262-w (PMC11790688; doi:10.1007/s12529-024-10262-w)
Supplement: Supplementary file 1 — Supplementary file1 (DOCX 19 KB) [file 12529_2024_10262_MOESM1_ESM.docx]

| **Supplemental-Table 1:** Search strategies according to the database until February 2023 |
| --- |
| **Web of Science** |
| **Intervention (1)** |
| TS=("psychotherapy" OR "psychol* intervention* " OR "psychosocial intervention*" OR "Health Education" OR "Patient Education" OR "Adaptation" OR "Coping" OR "intervention*" OR "Quantitative" OR "Treatment Outcome" OR "Psychometrics" OR "Empirical" OR "assessment" OR "psychopharmacology" OR "screening" OR "validation")  Limits: Articles; Topic |
| **Primary outcome (2)** |
| TS=(demoralization)  Limits: Articles; Topic |
| **Study design (3)** |
| TS= (randomized controlled trial OR controlled clinical trial OR randomization OR single blind procedure OR double blind procedure OR clinical trial OR quasi experimental study OR pretest posttest control group design OR intervention study OR pretest posttest design) |
| **In total, (1) AND (2) AND (3)** |
| Limits: Articles; Topic |
| **PUBMED** |
| **Intervention (1)** |
| psychotherapy OR psychol* intervention* OR psychosocial intervention* OR Health Education OR Patient Education OR Adaptation OR Coping OR intervention* OR Quantitative OR Treatment Outcome OR Psychometrics OR Empirical OR assessment OR psychopharmacology OR screening OR validation  Limits: No limit |
| **Primary outcome (2)** |
| Demoralization  Limits: No limit |
| **Study design (3)** |
| Randomized controlled trial OR controlled clinical trial OR randomization OR single blind procedure OR double blind procedure OR clinical trial/as topic OR quasi experimental study OR pretest posttest control group design OR pretest posttest design OR random*  Limits: No limit |
| **In total,** |
| (1) AND (2) AND (3) |
| **EMBASE** |
| **Intervention (1)** |
| 'psychotherapy '/exp OR 'psychosocial intervention'/exp OR ' Health Education '/exp OR 'Patient Education'/exp OR 'Adaptation'/exp OR 'Coping'/exp OR 'psychopharmacology '/exp OR 'Quantitative '/exp |
| **Primary outcome (2)** |
| Demoralization:ab,ti  Limits: Title or abstract |
| **Study design (3)** |
| 'randomized controlled trial'/exp OR 'controlled clinical trial'/exp OR 'randomization'/exp OR 'single blind procedure'/exp OR 'double blind procedure'/exp OR 'clinical trial'/exp OR 'quasi experimental study'/exp OR 'pretest posttest control group design'/exp OR 'pretest posttest design'/exp OR random* |
| **In total,** |
| (1) AND (2) AND (3) |
| **SCOPUS** |
| **Intervention (1)** |
| TITLE-ABS-KEY (psychotherapy OR "psychol* intervention* " OR "psychosocial intervention*" OR "Health Education" OR "Patient Education" OR Adaptation OR Coping OR intervention* OR Quantitative OR "Treatment Outcome" OR Psychometrics OR Empirical OR assessment OR psychopharmacology OR screening OR validation)  Limits: Title or abstract or keywords |
| **Primary outcome (2)** |
| ALL (demoralization) |
| **Study design (3)** |
| TITLE-ABS-KEY (“Randomized controlled trial” OR “controlled clinical trial” OR randomization OR “single blind procedure” OR “double blind procedure” OR “clinical trial” OR “quasi experimental study” OR “pretest posttest control group design” OR “pretest posttest design”) Limits: Title or abstract or keywords |
| **In total,** |
| (1) AND (2) AND (3) |
